# Secondary Histomorphological Changes in Cerebral Arteries of Normotensive and Hypertensive Rats following a Carotid-Jugular Fistula Induction

**DOI:** 10.1371/journal.pone.0092433

**Published:** 2014-03-19

**Authors:** Keith Ng, Masakazu Higurashi, Nahoko Uemiya, Yi Qian

**Affiliations:** 1 Australian School of Advanced Medicine, Macquarie University, Sydney, NSW, Australia; 2 Department of Neurosurgery, Yokohama Minami Kyousai Hospital, Yokohama, Japan; 3 Department of Endovascular Neurosurgery, Saitama Medical University, Saitama, Japan; University Medical Center (UMC) Utrecht, Netherlands

## Abstract

Haemodynamic changes in cerebral circulation are associated with the natural ageing process and associated pathology, leading to the development of incapacitating neurological and neurovascular diseases. Due to inherent biological limitations, current literatures mostly aimed at studying the correlation descriptively or quantifying the relationship in vitro or using computational models. In this paper, a model of a carotid-jugular fistula in the rat was used to create a haemodynamic insult to the intracranial arterial circulation and subsequent venous drainage. An arterial-venous (AV) fistula was created in 12 rats, 6 of which are normotensive Wistar-Kyoto strain (WKY) and the rest spontaneously hypertensive strain (SHR) with an additional 6 in each strains designed as controls without previous surgery. After 4 weeks of convalescence, all 24 rats were euthanised and their cerebral circulation was examined histomorphologically. We confirmed an intrinsic morphological difference between normotensive WKY and hypertensive SHR and found a modest but significant arterial shrinkage in both strains induced with AV fistula. We also reported that alterations in blood flow are also associated with marked extracellular matrix changes. We concluded that the model was suitable for studying the relative contributions of altering haemodynamic patterns and venous drainage on cerebrovascular changes. We also found that hypertension modulated cerebral vascular changes in addition to disrupted blood flow.

## Introduction

Chronic changes in cerebral haemodynamics are associated with morphological alterations in cerebral vasculatures [1]. The association can be observed in the presence of localised cerebral pathologies such as arteriovenous malformation [2], aneurysms [3], or under the effects of systemic haemodynamic insults such as hypertension [4], [5], ageing [6]–[8], or pathologies associated with ageing such as Alzheimer’s disease [9]. Cerebrovascular research has used a range of experimental models to unravel the causal relationship between haemodynamic changes and pathophysiological adaptation of cerebral vasculature in various diseases. Laboratory animal models enable researchers to take the correlation analysis often done in clinical trials a step further, from merely reporting a description of the coincidence of particular factors to providing a better understanding of the underlying mechanisms.

One of the many mechanical, myogenic, metabolic, neuronal, or biochemical parameters involved directly in modulating cerebral blood flow, leading to alterations in vascular resistance and cerebral blood flow is vascular dilatation [10]. Vascular dilatation has been shown to interact with cerebral metabolism and brain perfusion, contributing to a suboptimal cognitive function and various age-specific neurological dysfunctions [11]. To investigate the underlying pathophysiology, an experimental carotid-jugular fistula, for the purpose of altering cerebrovascular circulation, was used in the present study. An end-to-side anastomosis between the internal jugular vein and common carotid artery (AV fistula) was created. The fistula induces a focused persistent regional cerebral blood flow alteration by reversing blood drains into the venous system, shunting the perfusion pressure within the surrounding parenchyma and thereby, inducing structural adaptation of brain tissues to the artificial retrograde flow [12]. Disruption of cerebral circulation secondary to an AV fistula has been shown to greatly elevate the risk of developing co-morbidity, such as dysplastic aneurysms, in the presence of a previous or currently untreated cerebrovascular disease, leading to even higher mortality in cerebral vascular patients [13]. To our best knowledge, there is no spontaneously occurring cerebrovascular dilatation animal model available. The pathology of dilated cerebral vasculature is therefore, poorly understood. The aim of this study is to report a rat model of AV fistula that has incidental high occurrence of vascular dilatation in the circle of Willis proximal to the AV fistula and to further characterise the morphological and pathological features of the shrunk cerebral arteries. We further investigated the summative effects of hypertension in conjunction to altered haemodynamic changes on the vascular changes in the model.

## Materials and Methods

### Animals

6 normotensive WKY and 6 of their hypertensive counterpart SHR were used in this study. Hypertension status of SHR was confirmed by measuring their systolic blood pressure 1 week before experiments. Upon arrival at the laboratory, the rats were induced with general surgical anaesthesia with isoflurane (1–5% in 100% oxygen) and maintained with isoflurane or ketamine (75 mg/kg) in combination with an alpha adrenoceptor agonist medetomidine (0.1 mg/kg) both injected intraperitoneally. Rats were also given peri-operative antibiotics (caphazolin 200 mg) and analgesic (caprofen 0.1 mg/kg) intraperitoneally and a second dose of both medications given at 12 hours post surgery. Local anaesthetic (5% Lignocaine) was applied at the incision site. Depth of anaesthesia during surgery was assessed at 15 min intervals using withdrawal reflexes and their absence. Rats were then placed on a warm under sheet before surgery under sterile conditions began.

**Figure 1:**
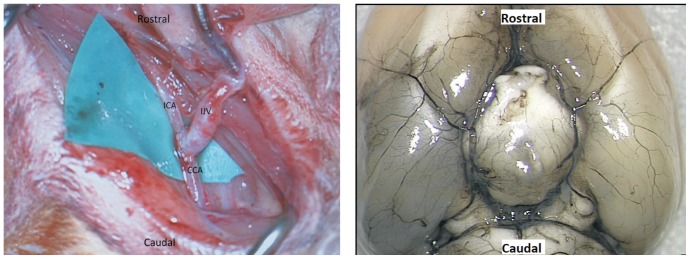
Left panel shows an AV fistula construction by anastomosing end-to-side left external jugular vein to common carotid artery. Post surgically, a reversed blood flow to the jugular vein was achieved. The vein was engorged by a higher blood pressure generated from the heart. Right panel shows the circle of Willis after fixation. Arteries were filled with TMD black dye by injecting the dye through punctuating the left ventricle of the heart after flushing the blood out completely with PBS. IJV: Internal jugular vein. ICA: Internal carotid artery. CCA: Common carotid artery.

Figure 1 shows an AV fistula immediately after induction (left panel) and the circle of Willis after fixation (right panel). To induce an AV fistula in animal, under magnification, the left external jugular vein (EJV) and the left common carotid artery (CCA) were exposed. An end-to-side anastomosis of the vein to the CCA was performed with a 10-0 nylon (Figure 1, left panel). This created a functional AV fistula between the circle of Willis and the left lateral venous sinus via a loop extension into the neck, composed of the internal carotid artery, distal common carotid artery, and the internal jugular vein. The fistula caused retrograde flow into the circle of Willis [14]. Although it was for a different cause, the model was well established and has been a model for the study of heamodynamic pathophysiology in human arteriovenous malformations [15]. Following surgery, rats were given atipamazole (0.05 ml, s.c.) to reverse the anaesthesia if ketamine and mededomidine were used. The animals were left on a warm pad during the recovery period. The animals were monitored every 15 min for 3 hours after the procedure. Once ambulatory, rats were returned to the animal housing facilities and continue to be monitored twice daily by experienced animal technicians. The animals were given further doses of NSAIDs for pain relief as needed in consultation with a licensed veterinarian. Rats were allowed to recover and housed at 24°C, exposed to 12 h of light and allowed free access to food and fluid. The Macquarie University Animal Ethics Committee approved all husbandry and experimental procedures.

### Histomorphometry

One month after the initial surgery, the rats were anesthetised with intraperitoneal pentobarbital sodium (30 mg/kg). Rats were perfused intra-arterially with Phosphate buffered saline. Each animal was hand injected with 2 ml black TMD tissue marking dye (Cole-Parmer Australia) into its circulation. Rats were then fixed with 4% neutral formalin.

Brain tissues were harvested and examined macroscopically immediately after fixation to detect the presence of any distal vascular changes (Figure 1, right panel). The patency of the AV fistula was confirmed in all rats by the presence of dye in the AV fistula and direct visual investigation of the AV fistula. Diameter of the vessel wall in the circle of Willis was measured through an operating microscope interfaced with the image analysing system Image J [16]. The brain was then embedded in paraffin, and sectioned longitudinally at 50 μm thick sections, both at the vessel proximal to the AV fistula (left cerebral arteries) and their contralateral sites (right cerebral arteries). The tissues were stained with hematoxylin and eosin for visualisation of histological structures. Diameters of internal carotid artery, middle cerebral artery, and anterior cerebral artery were measured and compared between strains. Volume densities of smooth muscle, elastin, basement membrane, and endothelium were qualitatively examined from histological sections interfaced with image processing software Image J. Distribution and fragmentation of elastin lamellae were reported descriptively. Diameters were measured again on histological section under light microscope and measurements were repeated five times at different locations before an average was reported. The histological analysis method has been extensively used in our group with good reproducibility.

### Statistical Analysis

Results were first compared qualitatively. Changes in volume densities of smooth muscle, elastin, basement membrane and endothelium from histological sections were examined and compared. Arterial shrinkage in terms of diameters was statistically compared between strains pre and post surgical AV fistula induction with one-way ANOVA and post-hoc Bonferroni correction. All differences were considered significant when *P<0.05*.

## Results

### Cerebral Arterial Changes

All of the rats survived the surgical procedures, lost weight post-operatively but gradually regained their weight during and after convalescence. We ruled out the effect of fixation resulting in significant shrinkage of cerebral arteries by standardising all fixation procedures across all rats and we also used contralateral arteries as a means of comparison. Although variability in cerebral arteries was anticipated, our results confirmed that such variation was not significant (Table 1).

**Table 1:** Morphological and haemodynamic characteristics of cerebral arteries in sham and AV fistula rats.

	Sham				Fistula			
	WKY		SHR		WKY		SHR	
	Right	Left	Right	Left	Right	Left	Right	Left
Age, weeks	36±4				36±4			
Weight, g	428±7				392±21			
Systolic blood pressure, mmHg	116±15		178±22*		108±14		165±25*	
External diameter, μm								
ICA	245±10	230±8	157±33^#^	151±37^#^	220±42	170±36†	114±40^#^	102±22^#^ †
MCA	165±4	157±9	118±30^#^	119±33^#^	115±44	127±24†	76±30^#^	73±21^#^ †
ACA	176±16	169±8	95±24^#^	92±19^#^	132±45	116±21†	64±23^#^	59±23^#^ †

ICA: internal carotid artery. MCA: middle cerebral artery. ACA: anterior cerebral artery.

*^#^indicate significant different from WKY of the same group (sham or fistula).

† indicates significant different from sham groups. *P<0.05.*

Table 1 shows the characteristics of rats and the parameters measured in the study. As expected, SHR showed significantly higher systolic blood pressure measured by tail-cuff compared with WKY. The left and the right cerebral arteries, including internal carotid artery (ICA), middle cerebral artery (MCA), and anterior cerebral artery (ACA) showed no significant difference bilaterally in their dimensions in sham rats. Sham SHR showed smaller cerebral arteries compared with WKY.

**Figure 2:**
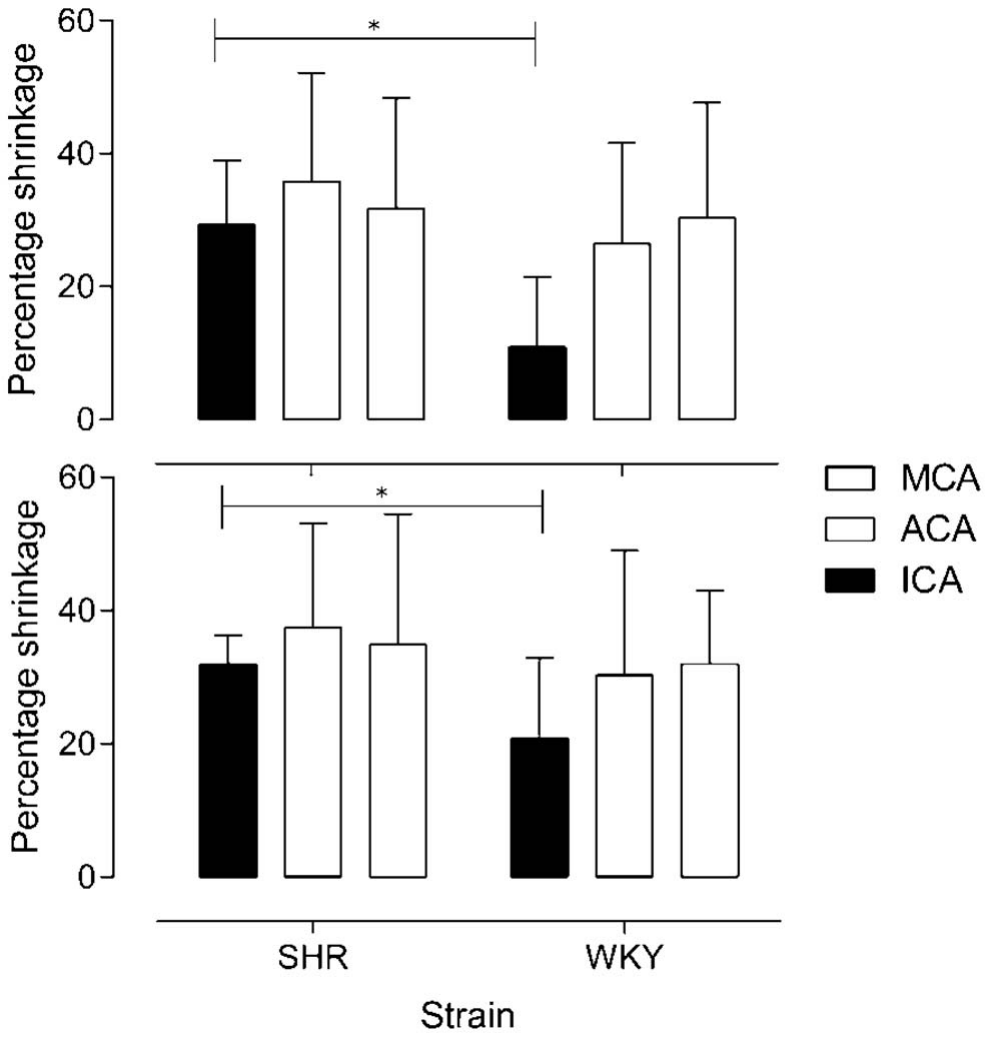
Shrinkage of cerebral vessels before and after AV fistula. Rats were sacrificed 4 weeks post operated and shrinkage was calculated from a percentage between fistula group and their respective controls. Top panel shows right arteries and bottom panel shows the left. Values are mean±SD. * indicates statistical significance. *P<0.05*.

The diameter of the arteries in the circle of Willis was considerably smaller in rats with fistula than those without (Figure 2). WKY started with larger vessels measured in diameter and showed a greater shrinkage in absolute values. In comparison, SHR shrank to a greater degree in terms of percentage shrinkage. Left vessels showed greater shrinkage post-operatively compared with the right. Although surgical procedures were well tolerated by all rats, SHR developed significantly more complications such as retina haemorrhages (n = 3) and significant weight loss (>5%, n = 2) following successful AV fistula induction.

### Histomorphological Parameters

**Figure 3:**
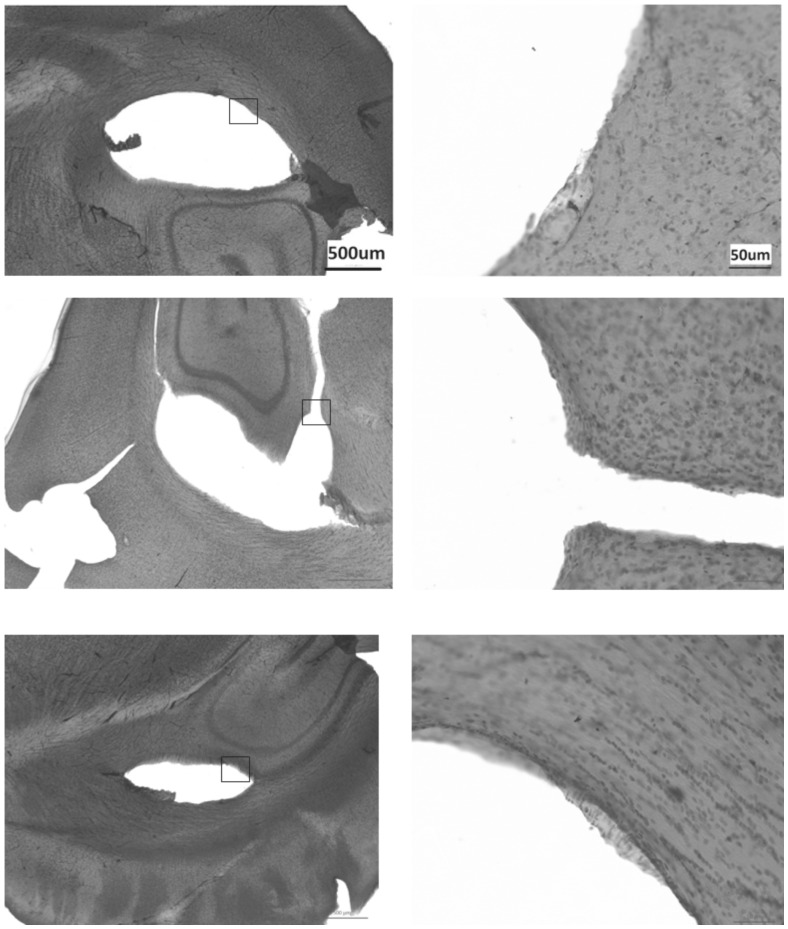
Representative histological sections at respective arteries of sham SHR. Top panel: ICA, middle panel: MCA and bottom panel: ACA. Left pictures were taken at 10x magnification. Squares in the left panel indicate ROI magnified on the right magnified at 40x. All images follow the same scale bar of the image in their corresponding top panel.

**Figure 4:**
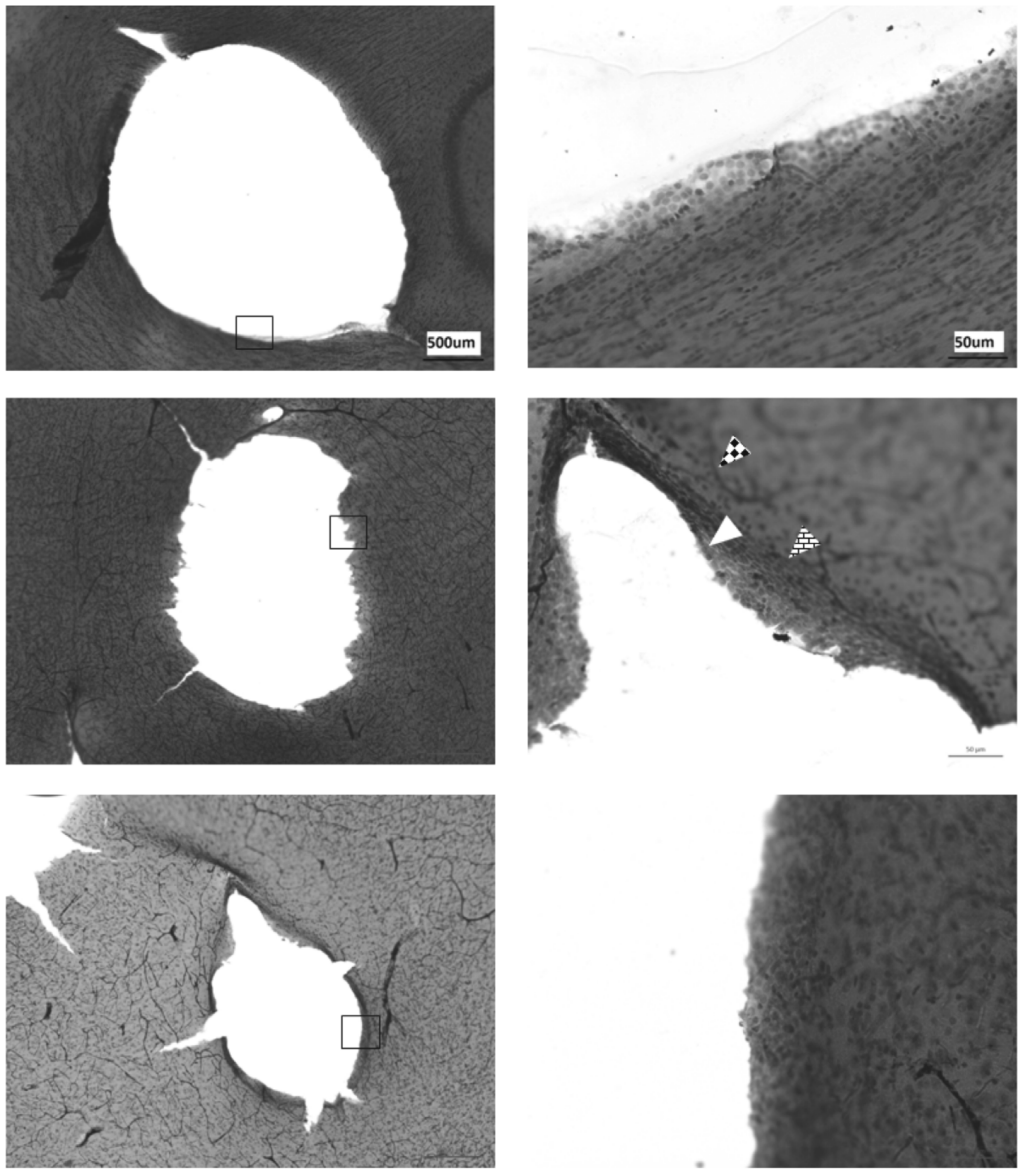
Representative histological sections at respective arteries of fistula SHR. Top panel: ICA, middle panel: MCA and bottom panel: ACA. Left pictures were taken at 10x magnification. Squares in the left panel indicate ROI magnified on the right magnified at 40x. Unfilled triangle shows endothelium. Diamond filled triangle shows elastin lamellae. Brick filled triangle shows basement membrane. All images follow the same scale bar of the image in their corresponding top panel.

The morphology of the vessel wall in sham and fistula rats is shown in Table 1. Representative vessel structure was stained with hematoxylin and eosin (WKY in Figure 3 and SHR in Figure 4). Both sham and fistula SHR showed greater volume densities of smooth muscle cells and elastin lamellae compared with their respective shams. Comparing fistula SHR (Figure 4) to fistula WKY (Figure 3), the difference was further widened in fistula SHR, indicating greater preservation in extracellular matrix in SHR than in WKY. In both groups of rats, the left cerebral arteries showed greater increase in extracellular matrix compared with farther right side. Both basement membrane and endothelium appeared more prominent and organised in fistula rats. Thus, induction of AV fistula increased composition of left cerebral arteries in fistula rats but to a lesser extent in the right vessels. The difference in left and right extracellular matrix composition was not observed in sham rats. Qualitatively in fistula rats, a marked disruption of elastin lamellae can be observed in the areas surrounding the vessel. A marked disorganisation of smooth muscle cells and endothelium were also observed.

## Discussion

There are three major findings in this study. First, we reported that a rat model of carotid-jugular fistula commonly used to study arteriovenous malformations developed significant cerebral arterial shrinkage in the circle of Willis. Second, following AV fistula induction, SHR developed greater shrinkage compared with WKY and the left ICA, MCA and ACA directly proximal to the AV fistula shrank more compared with their contralateral side. Thirdly, the shrinkage was associated with a proportionate increase in extracellular matrix hypertrophy and smooth muscle cells, a more organised and less fragmented elastin network, and a less disruption in basement membrane and endothelium.

### Evaluation of the Model

One of the goals of this study was to characterise changes in cerebral arteries under the influence of haemodynamic insults following AV fistula. We studied the effects in two strains, the normotensive WKY and the hypertensive SHR. We hypothesised that cerebral arteries in both WKY and SHR adapt to changes in cerebral haemodynamic and exposing rats to higher blood pressure may accentuate any structural alteration.

Idiopathic dilatation of cerebral vessels or pathological changes in the cerebral arterial wall that result in neurological deficits or haemorrhage were rare in animals [17] and it is unlikely to induce an AV fistula directly in the rat brain due to extreme technical difficulty. Despite that the fistula we created was located in the neck, because of the close proximity of the CCA to the circle of Willis, a location prone to development of vascular pathologies, we made an assumption that the altered mechanics and structure in vasculature directly proximal to the AV fistula is likely to be resulted from the fistula.

In addition to some of the previous studies that have used similar AVM model for some other purposes, we demonstrated for the first time that they are histomorphological changes in the cerebral vessels away from where the AV fistula was induced. This is a critical distinction and an important finding because existing models of infarcts in the literature focused mostly on creating ischaemia by blocking the vessels with emboli and the lesions created were mostly too large, affecting a large portion of the cortex [18]–[20]. Few models mimicked small vessel pathology and, to our best knowledge, not one without causing blood-brain barrier failure [21]. We showed, in our model, histomorphological changes in the cerebrovasculature that are directly relevant and would aid understanding of the etiology and help develop treatments for stroke due to failure in vascular integrity.

Another consideration with respect to the validity of the model is the relatively short period of time between creation of AV fistula and the initiation of vascular changes in our model. Naturally, in humans, alteration in cerebrovascular structure and function is likely to be chronic, taking possibly decades to develop. We assumed that the risk for the development of a secondary cerebral pathology was greatly increased in humans or animals with a primary untreated AV fistula. Even so, for reasons that are still unknown, a subpopulation of cerebrovascular patients may not develop a secondary vascular pathologies despite the disrupted mechanics, and for those who do, the dilatation may not be symptomatic or pose any significant damage to the patients [13]. We chose one month as the time between creation of AV fistula and examination of cerebral arteries because, based on our experience, one month is a reasonable incubating period for sufficient cerebrovascular changes with fewest of the rats developing significant neurological deficit or cerebral haemorrhage detrimental to their well-being before euthanasia. Considering the trend we observed, even in a short period of time, we can reasonably extrapolate that, when left longer, the cerebral pathologies are likely to worsen and thus, the association could be even stronger. Besides, our approach avoided some of the disadvantages of altering cerebral vessel changes pharmacologically, which may confound our result by an additional neuronal and humoral effect at the systemic level [22].

### Consideration of Previous Studies

Our study focused on relatively larger arteries immediately proximal to the fistula and we found that ICA, MCA, and ACA shrank following AV fistula with vessels of SHR shrank more compared with WKY. We postulate that this observation is likely due to the intrinsic distensibility of cerebral arteries in these rats, a concept we will explain in further details in the following paragraphs. There have been controversies in the observation; studies in humans showed that arteries adjacent to AV fistula dilate resulted from thinning of the vessel wall, thus increased distensibility, whereas veins become arteriolised with hypertrophy of smooth muscle cells on the wall [23]. Evidence to the contrary has also been reported. Studies in animal showed dilatation of arterial wall is not always the case, dependent on exposure time and location of the AV fistula [24]. Intuitively speaking, thinner wall gives higher probability for uncontrollable further dilatation leading to rupture, although it is not necessarily true [25]. Regardless, changes in the cerebral circulation are likely to resemble a vicious cycle - the arterial wall remodels following haemodynamic insults, which subsequently, accentuate further remodelling. To date, the exact mechanism is still largely unknown [26]–[28].

### Determinants of Morphological Changes Following AV Fistula

Morphological changes resulted from altered haemodynamics have been linked to a variety of physiological modulations, including changes in pressure and flow patterns, humoral effects, neural effects, and genetic determinants. This paper focused on the morphology of cerebral arteries in association with haemodynamic changes. We found that both WKY and SHR showed significant cerebral vessel shrinkage following AV fistula further support a direct relationship between the two. Although the feeding pressure and venous outflow were not measured in this study, an AV fistula would induce both a significantly higher feeding pressure to the venous system via jugular vein and the reversed flow will result in increased venous outflow resistance, insinuating flow in the left cerebral arteries, which may obliterate the predisposition to enlargement.

Although our results showed that cerebral vessels shrank in both WKY and SHR, we showed that SHR shrank to a greater extent compared with WKY measured by a percentage between the diameters of cerebral vessels of the fistula rat to that of the sham rats. This may appear counter intuitive at first. Considering previous findings that showed the larger cerebral arteries of SHR rats, such as those examined in this study, were significantly stiffer with a reduction in vessel external diameter compared with WKY [29], and since SHR had higher blood pressure, one can then postulate that a higher reversed flow was achieved after AV fistula, leading to lower pressure in the arteries proximal to the AV fistula compared with sham SHR. Under a greater reduction in distending pressure, SHR was expected to regress further. Our observation in WKY further supports the postulation. In absolute terms, although not statistically significant, cerebral vessels of the WKY shrank more but because WKY had larger diameter at the start, its percentage shrinkage was less than that of SHR.

### Morphological Changes and Composition

As with other previous studies that proved a correlation between morphological changes in the vessel wall and surrounding compositions following haemodynamic insults [30]–[32], our results support that a disturbed cerebral circulation was associated with a proportional composition alteration of the extracellular matrix. In this study, we directly measured volume densities of elastin content, smooth muscle cells, and endothelium as relatively distensible components and compared them against basement membrane, which we regarded as relatively non-distensible. Although we did not measure collagen directly, we argue that the density of collagen is extremely low in brain tissues in rats and not only that it required electron microscopy for an accurate measurement, but also most of the collagen is contained mostly in the basement membrane [17]. By examining the basement membrane, we could reasonably deduce the resulted effect from combined changes on morphology and vessel function. Interestingly, similar changes were also observed in cerebral arteries in ageing humans [33] and animals [34]. A study on older senescent rats showed that rats over 24 months old developed cerebral arteries with smaller diameters and reduced distensibility due to a reduction in the collagen/elastin ratio [35]. Under the interaction of several variables, including intravascular pressure and lumen diameter, a complex pattern of initial outward hypertrophy, also demonstrated in this study, followed by an inward hypertrophic remodelling can be observed. Hypertrophy of the extracellular matrix may contribute to increased intrinsic vascular tone [36], an association that has been shown in with stiffer resistant arteries [37]. These observations, together with ours, underscore the importance of interaction between extracellular matrix changes and pathology in determining cerebral vascular function.

We confirmed that, in sham animals, SHR showed greater density in all extracellular matrix compositions compared with WKY. We also showed post-operatively, SHR experienced greater increase in all components, which led us to speculate that SHR became more distensible following AV fistula. Both strains also showed a more organised elastin matrix with reduced fragmentation. This observation has important physiological significance. SHR has been chronically exposed to higher blood pressure and its cerebral vessels have been shown to be stiffer [29]. Presumably, the adaptation is as a result of greater haemodynamic insults from central circulation. Stronger vessels improve the ability of the blood vessels to withstand pulsatile energy from the central circulation, thus protects the brain from end-organ damage.

## Conclusions

The findings in this study show that rats induced with an AV fistula could also develop concomitant secondary cerebral vascular pathology proximal to the fistula. We observed that both normotensive and hypertensive rats had equal potential in developing the pathology. When haemodynamic changes were present, we found that hypertension accentuated the pathology, causing not only more severe shrinkage but also more complications. To understand the interaction of the two seemingly independent but closely related pathologies, development of a cost effective, relatively easy to prepare animal model will likely be useful. We observe that hypertension *per se* is not as effective as when combined with AV fistula in augmenting the likelihood of developing vascular changes. Because the intrinsic differences in structure and function of cerebral vessels in WKY and SHR, comparisons or interpretations of the results made by this model should be done with cautions especially when inferring the result to observations in humans. The simplest interpretation of our data is that haemodynamic insults drive both the vascular wall and extracellular matrix adaptation, whose main function is to regulate cerebral blood flow, thereby protect the brain from hypo- or hyper-perfusion under conditions of severe haemodynamic insults from systemic pressure. Although the role of hypertension in cerebral arterial pathology is still not completely understood, chronic exposure to cyclic strain may result in realignment of vascular smooth muscle cells in a pattern that further compromises vascular integrity. Any attempt to keep blood pressure in check is likely to be useful clinically.
